# Efficacy and safety of antibody-drug conjugate combination therapy in advanced urothelial carcinoma

**DOI:** 10.3389/fonc.2025.1669526

**Published:** 2025-10-07

**Authors:** Salvador Jaime-Casas, Regina Barragan-Carrillo, Miguel Zugman, Koral Shah, Hedyeh Ebrahimi, Benjamin Mercier, Daniela V. Castro, Peter D. Zang, Alexis LeVee, Wesley Yip, Xiaochen Li, Nazli Dizman, Nicholas J. Salgia, Zeynep Zengin, Luis Meza, JoAnn Hsu, Charles B. Nguyen, Alexander Chehrazi-Raffle, Sumanta K. Pal, Abhishek Tripathi

**Affiliations:** ^1^ Department of Medical Oncology & Experimental Therapeutics, City of Hope Comprehensive Cancer Center, Duarte, CA, United States; ^2^ Division of Urology and Urologic Oncology, City of Hope Comprehensive Cancer Center, Duarte, CA, United States; ^3^ Department of Hematology and Oncology, MD Anderson Cancer Center, Houston, TX, United States; ^4^ Department of Immunology, Roswell Park Comprehensive Cancer Center, Buffalo, NY, United States; ^5^ Department of Internal Medicine, Yale University School of Medicine, New Haven, CT, United States

**Keywords:** urothelial carcinoma, antibody-drug conjugate, clinical trials, immunotherapy, nectin-4, Trop-2, HER2

## Abstract

Antibody-drug conjugates (ADCs) are revolutionizing the treatment landscape of advanced urothelial carcinoma (aUC). We systematically reviewed the PubMed and Embase databases for published clinical trials evaluating ADC-combination regimens in aUC. We extracted safety and efficacy outcomes, including objective-response rate (ORR), adverse events (AEs), and ≥ grade 3 AEs. We excluded narrative reviews, retrospective studies, and case reports. Two independent reviewers screened titles and abstracts for relevance, followed by a full-text review for eligibility. A total of 645 patients from 5 trials investigating anti-nectin-4 (enfortumab vedotin, EV), anti-TROP2 (sacituzumab govitecan, SG), and anti-HER2 (disitamab vedotin, DV) ADCs were identified. We recorded a pooled ORR of 65%. We recorded a pooled risk rate for all-grade toxicity of 57%. The most prevalent any-grade AEs were peripheral sensory neuropathy 52% (95% CI, 45%-59%), fatigue 45% (95% CI, 28%-64%), and diarrhea 42% (95% CI, 16%-74%). Peripheral sensory neuropathy, fatigue, and alopecia were more commonly observed in anti-nectin-4 regimens. Gastrointestinal (diarrhea and nausea) and hematologic (anemia and neutropenia) toxicities were more commonly observed in anti-TROP2 regimens. Hepatotoxicity was predominantly found in anti-HER2 regimens. While ADC-based combination regimens show promising responses, they also have high rates of AEs in patients with aUC.

## Introduction

Antibody-drug conjugates (ADCs) targeting nectin-4, trophoblast cell-surface antigen 2 (TROP2), and human epidermal growth factor receptor 2 (HER2) have shown efficacy as monotherapy in advanced urothelial carcinoma (aUC) (1–3). Multiple trials have combined immune checkpoint inhibitors (ICIs) and ADCs (NCT06823427, NCT06483334, NCT05911295) (3–7). Notable ADCs in this space include enfortumab vedotin (EV) (anti-nectin-4 antibody with a monomethyl auristatin E (MMAE) payload and a cleavable linker), sacituzumab govitecan (SG) (anti-TROP2 antibody with a SN-38 payload and a cleavable linker), and disitamab vedotin (DV) (anti-HER2 monoclonal antibody with a MMAE payload and cleavable linker). With the approval of EV with pembrolizumab in untreated aUC and several ongoing trials of ADC/ICIs, most patients are likely to receive ADC-based combination therapy as part of their treatment in the future. However, considering the different therapeutic target antigens and payloads (microtubule vs. topoisomerase inhibition) of ADCs, these combinations can have varying efficacy and tolerability. We performed a systematic review and meta-analysis on the efficacy and toxicity of anti-nectin-4, anti-TROP2, and anti-HER2 ADC-based combinations in aUC.

## Methods

We searched Embase and PubMed bibliographic databases through May 2025 in accordance with the Preferred Reporting Items for Systematic Reviews and Meta-analyses (PRISMA) guidelines (**Supplementary Figure 1**). Prospective clinical trials reporting efficacy and tolerability of ADC combination therapy (ADC + ICI) in patients with aUC were included. We excluded case reports, retrospective cohorts, and narrative reviews. Two independent reviewers (SJC and RBC) examined titles and abstracts for inclusion. Discrepancies were resolved through unanimous agreement (AT). Studies were grouped into anti-TROP2, anti-nectin-4, and anti-HER2 combinations. The pooled objective response rate (ORR) and adverse event (AE) rates with 95% confidence intervals (CIs) were calculated using a random-effects model. Interstudy heterogeneity was analyzed using the Chi-square test and I2 statistics. Heterogeneity was defined as low (I2 < 30%), moderate (30% < I2 < 70%), and high (I2 > 70%). For outcomes with moderate heterogeneity and higher, a random-effects model was used. Otherwise, a fixed-effect model was used. We performed descriptive analysis if data could not be combined. Statistical analysis was conducted using R Statistical Software (v4.3.3; R Core Team 2024). Significance was defined as two-sided (p < 0.05).

## Results

**Figure 1:**
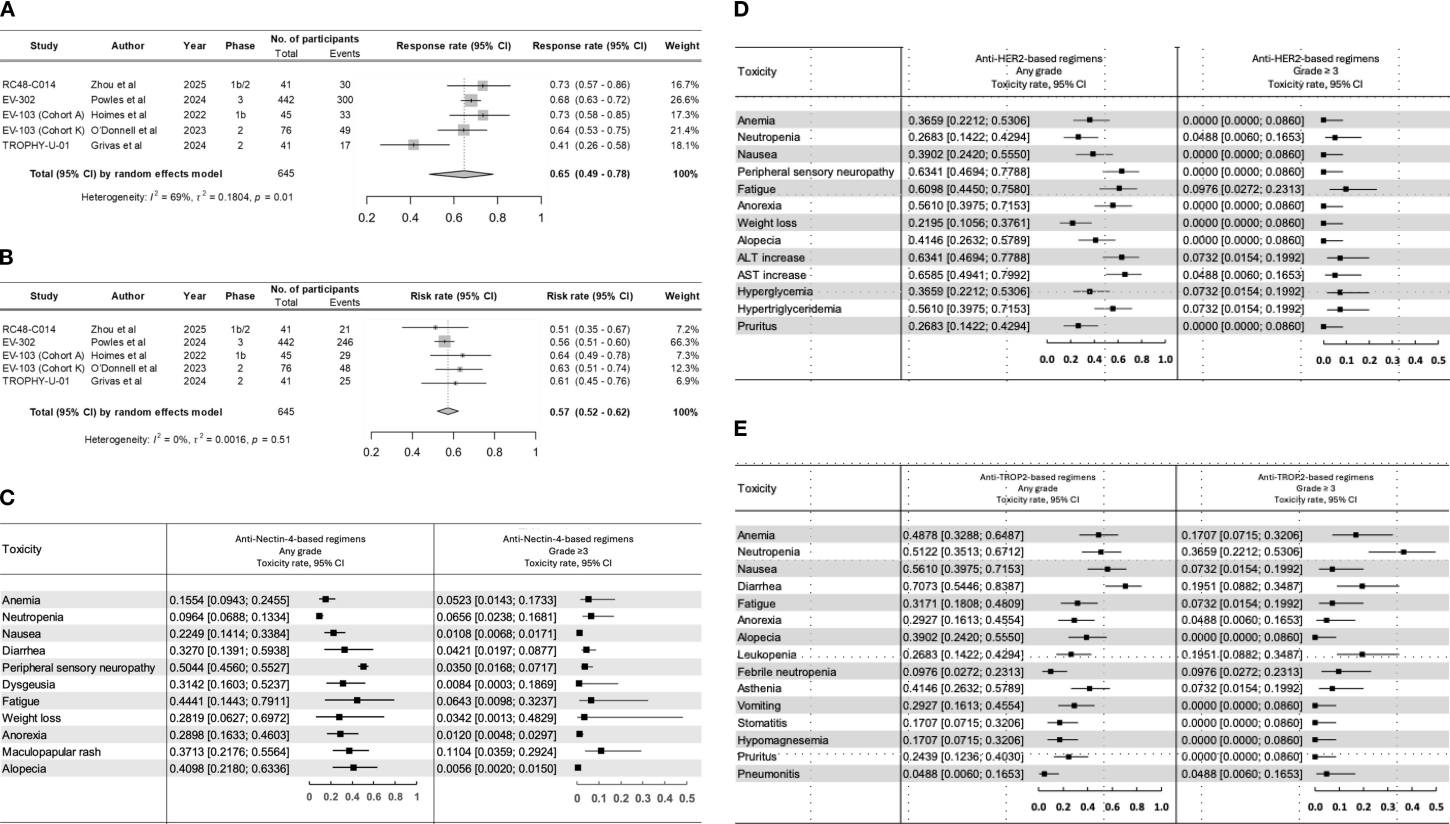
Pooled rate of **(A)** objective response and **(B)** toxicity across clinical trials across clinical trials. Forrest plots for all grade and grade ≥3 adverse events observed with **(C)** anti-nectin-4, **(D)** anti-HER2, and **(E)** anti-TROP2 combination regimens.

After screening, five studies were eligible for data extraction (3, 8–11) (**Table 1**). These included the TROPHY-U-01 (Cohort 3), EV-302, EV-103 (cohort K), and EV-103 (cohort A), and RC48-C014 trials (3, 8–11). Drugs evaluated across these trials included EV (EV-302, EV-103 cohort K, and EV-103 cohort A), SG (TROPHY-U-01 Cohort 3), and DV (RC48-C014). The pooled ORR was 65% (95% CI, 49%–78%). There was significant heterogeneity noted between trials (I^2^ = 69%; p = 0.01), largely driven by differences in efficacy between anti-nectin-4, anti-HER2, and anti-TROP2 combinations. For anti-nectin-4 trials, ORR ranged from 73% (EV-103; cohort A: 95% CI, 58%-85%) to 68% (EV-302: 95% CI, 63%-72%) and 64% (EV-103; cohort K: 95% CI, 53%-75%). The anti-HER2 ADC RC48-C014 trial showed an ORR of 73% (95% CI, 57%-86%; **Figure 1A**) while the TROPHY-U-01 trial showed an ORR of 41% (95% CI, 26%-58%).

**Table 1:** Summary of included clinical trials.

Study name	Phase	Disease setting	Total sample size (n)	Combination group sample size (n)	Control group (if available) sample size (n)	Efficacy in combination group	Rate of grade 3 or above AEs	TRAEs leading to dose reduction	TRAEs leading to dose discontinuation	TRAEs resulting in death
RC48-C014	Phase Ib/II	Locally advanced or mUC.	41	41	NA	ORR 73% (95% CI, 57-86)	51.2%	36.6%	14.6%	1 patient
EV-302	Phase III	Locally advanced or mUC.	886	442	444	OR 67.7% (95% CI, 63.1-72.1)	55.9%	40.7%	35%	4 patients
EV-103, Cohort K	Phase Ib/II	Treatment naive locally advanced or mUC.	51	76	73	cORR 64.5% (95% CI, 52.7-75.1)	63.2%	NA	47.4%	3 patients
EV-103 Cohort A	Phase Ib/II	Locally advanced or mUC.	45	45	NA	ORR 73.3% (95% CI, 58.1-85.4).	64.4%	31.1%	24.4%	1 patient
TROPHY-U-01 Cohort 3	Phase II	mUC	41	41	NA	ORR 41% (95% CI, 26.3-57.9).	61%	39%	15%	No patients

ECOG, Eastern Cooperative Oncology Group; ICI, immune checkpoint inhibitor; OR, overall response; ORR, objective response rate; cORR, confirmed objective response rate; mUC, metastatic urothelial carcinoma, TRAEs, treatment related adverse events.

We recorded a pooled risk rate for all-grade toxicity of 57% (95% CI, 52%-62%) without significant interstudy heterogeneity (I^2^ = 0%; p = 0.51) (**Figure 1B**). The highest risk was observed in the EV-103 (cohort A), 64% (95% CI, 49%-78%), and EV-103 (cohort K), 63% (95% CI, 51%-74%) trials. Overall, the most prevalent any-grade AEs were peripheral sensory neuropathy 52% (95% CI, 45%-59%), fatigue 45% (95% CI, 28%-64%), and diarrhea 42% (95% CI, 16%-74%). (**Supplementary Table 1**). The incidence of AEs varied significantly between regimens. The most common any-grade AEs in anti-nectin-4 regimens included peripheral sensory neuropathy 50% (95% CI, 45%-55%), fatigue 44% (95% CI, 14%-79%), and alopecia 40% (95% CI, 21%-63%). Grade ≥3 AEs included maculopapular rash 11% (95% CI, 3%-29%) and neutropenia 6% (95% CI, 2%-16%). The most common any-grade AEs in anti-TROP2 regimens were diarrhea 70% (95% CI,54%-83%), nausea 56% (95% CI, 39%-71%), and neutropenia 51% (95% CI, 35%-67%). Grade ≥3 AEs included neutropenia 36% (95% CI, 22%-53%) and diarrhea 19% (95% CI, 8%-34%). The most common any-grade AEs in anti-HER2 regimens were AST increase 65% (95% CI, 49%-79%), ALT increase 63% (95% CI, 46%-77%), and peripheral sensory neuropathy 63% (95% CI, 46%-77%). Grade ≥3 AEs included fatigue 9% (95% CI, 2%-23%), ALT increase 7% (95% CI, 1%-19%), and hyperglycemia 7% (95% CI, 1%-19%). (**Figures 1C–E**).

## Discussion

Our study highlights the significant heterogeneity in the efficacy and toxicity profile of ADC-based combination regimens in aUC. Differential expression of the target antigen and the therapeutic design of each drug likely drive the significant heterogeneity in efficacy and toxicity profiles. Peripheral sensory neuropathy, fatigue, and alopecia were more commonly observed in anti-nectin-4 regimens, consistent with other Monomethyl Auristatin E (MMAE) containing ADCs (brentuximab vedotin, glembatumumab vedotin, and polatuzumab vedotin) (12). This on-target off-tumor toxicity is due to the high expression of nectin-4 in the skin and the effect of the MMAE payload (12). Peripheral neuropathy is also associated with ADCs composed of tubulin inhibitor payloads (MMAE, SPP-DM1, and SPDB-DM4) and is caused by the peripheral axonopathy induced by free payload release into the systemic circulation (12). The hepatotoxicity described in the RC48-C014 trial likely reflects HER2-dependent and independent pathways, resulting from the normal expression of HER2 in hepatocytes and the internalization of disitamab vedotin (12). In contrast, gastrointestinal (diarrhea and nausea) and hematologic (anemia and neutropenia) toxicities were more common in the TROPHY-U-01 (Cohort 3) trial and are mediated by the release of SN-38 to the systemic circulation (12). Of note, hematological suppression can be so profound that a black box warning was added to SG for severe or life-threatening neutropenia and severe diarrhea. This echoes the results of the recent TROPiCS-04 study, where SG failed to meet the primary endpoint of overall survival compared to chemotherapy, despite higher ORR (23% vs. 14%) and progression-free survival (4.2 vs. 3.6 months) (13). Notably, the development of severe neutropenia and failure of the TROPiCS-04 study to meet its primary endpoint resulted in Gilead Sciences voluntarily withdrawing SG’s indication for treatment of adult patients with pretreated, locally advanced, or metastatic urothelial carcinoma. This could lead to fewer trials being developed in the pipeline of SG-related combinations and more efforts being made for other anti-TROP2 ADCs. This further underscores the safety signals across all other trials, specifically for anti-nectin-4-based trials, which showed the highest rates of treatment-related adverse events (TRAEs) leading to regimen modifications. For example, the EV-302 trial reported a dose reduction rate of 40.7% and 4 deaths due to TRAEs, and the EV-103 (cohort K) trial reported a treatment discontinuation rate of 47.4% and 3 deaths due to TRAEs.

Although the indication for SG in aUC was withdrawn, many other anti-TROP2 ADCs are currently in development as monotherapy and in combination. Datopotamab-deruxtecan (Dato-DXd) demonstrated an ORR of 25% in pre-treated patients, with no grade ≥ 3 hematologic events reported (14). Sacituzumab tirumotecan, an anti-TROP2 ADC with a topo-1 inhibitor payload, is currently being investigated in combination with EV with or without pembrolizumab in the phase 1/2 KEYMAKER-U04 trial (NCT06483334). Advances in the design of ADCs could also improve the safety and tolerability of anti-nectin-4 ADCs. BT8009 is a bicycle toxin conjugate targeting nectin-4 with an MMAE payload linked via a valine-citrulline cleavable linker (15). Preliminary results of the Duravelo-2 study (NCT06225596) showed an ORR of 45% with no grade ≥3 adverse events reported (15).

In the current treatment landscape, EV in combination with pembrolizumab is considered the standard of care for first-line treatment of advanced or metastatic urothelial carcinoma following the promising results of the EV-302 trial, which demonstrated an objective response rate of 67.7% (3). Our pooled analysis of ADC-based combinations (including anti-TROP2, anti-nectin-4, and anti-HER2 ADCs) yielded a similar efficacy to that of EV plus pembrolizumab alone, though at the cost of distinct toxicity profiles. These findings suggest that ADC-based combination regimens may consolidate their role as alternative strategies to EV plus pembrolizumab in the frontline setting, while also remaining alternative options in the salvage setting. However, their definitive role will need to be clarified through ongoing and future randomized trials.

Our study has some limitations. First, the number of eligible trials was small, and pooled subgroup analyses were not feasible for anti-TROP2 and anti-HER2-based regimens, since each was represented by a single study. We therefore reported regimen-level results descriptively, while retaining an overall pooled analysis across all ADC combination regimens. Second, the variability in trial design, patient population, and eligibility criteria may have contributed to interstudy heterogeneity. Moreover, the predominance of anti-nectin-4-based trials limits the generalizability across all ADC drug classes. As the therapeutic landscape in advanced urothelial carcinoma is rapidly evolving, forthcoming trial results are likely to refine the role of ADC-based combinations.

Combination approaches incorporating ADCs have changed the current treatment landscape of aUC, with several studies currently underway. While the non-overlapping toxicity profile of ADCs favors combination strategies, careful assessment of the cumulative risk is crucial for the development and safe clinical adoption of these combinations.

## Data Availability

The raw data supporting the conclusions of this article will only be shared upon reasonable request to the corresponding author.

## Supplementary material

Supplementary Figure 1 Article selection process (PRISMA).

Supplementary Table 1 Most common any grade and grade 3 or above adverse events across all studies.

Supplementary Table 2 Systematic review search syntax for PubMed and Embase online libraries.
