# Supplementary figures and images for: Efficacy and safety of antibody-drug conjugate combination therapy in advanced urothelial carcinoma
Source: Front Oncol. 2025 Oct 7;15:1669526. doi: 10.3389/fonc.2025.1669526 (PMC12537358; doi:10.3389/fonc.2025.1669526)

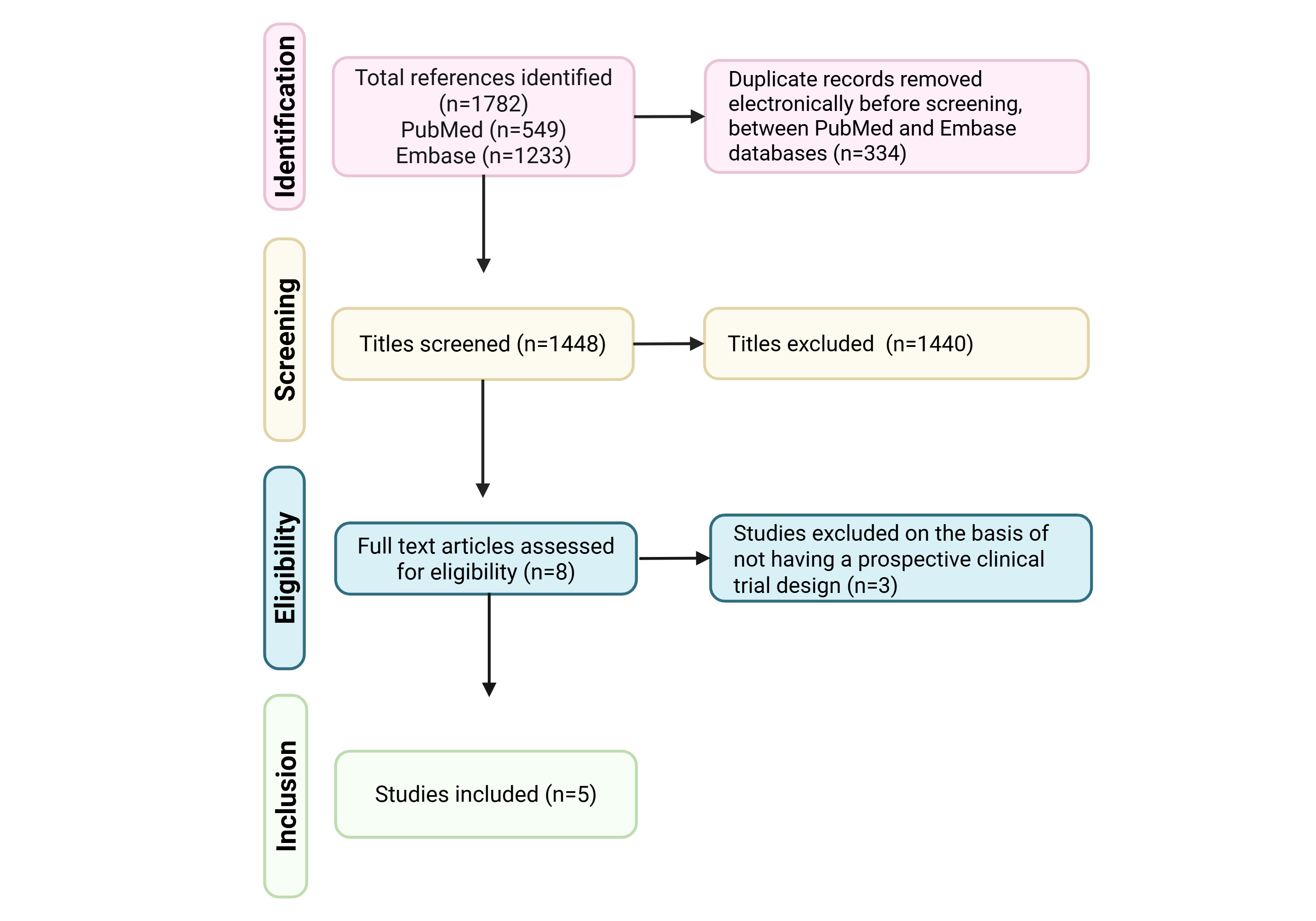

Supplement: Supplementary Figure 1 — Article selection process (PRISMA). [file Image1.jpeg]
