# Supplementary material for: Efficacy and safety of antibody-drug conjugate combination therapy in advanced urothelial carcinoma
Source: Front Oncol. 2025 Oct 7;15:1669526. doi: 10.3389/fonc.2025.1669526 (PMC12537358; doi:10.3389/fonc.2025.1669526)
Supplement: Supplementary Table 1 — Most common any grade and grade 3 or above adverse events across all studies. [file Table1.docx]

**Supplementary table 1. Most common any grade and ≥ grade 3 adverse events across all studies**

| **Toxicity** | **Any Grade** | **Grade≥3** |
| --- | --- | --- |
|  | **Risk rate, 95% CI** | **Risk rate, 95% CI** |
| **Diarrhea** | 0.42 [0.16; 0.74] | 0.07 [0.02; 0.23] |
| **Peripheral sensory neuropathy** | 0.52 [0.45; 0.59] | 0.03 [0.02; 0.06] |
| **Fatigue** | 0.45 [0.28; 0.64] | 0.07 [0.03; 0.14] |
| **Alopecia** | 0.40 [0.32; 0.49] | 0.007 [0.004; 0.01] |
| **Maculopapular rash** | 0.37 [0.22; 0.56] | 0.11 [0.04; 0.29] |
| **Nausea** | 0.32 [0.18; 0.51] | 0.02 [0.005; 0.07] |
| **Anemia** | 0.25 [0.12; 0.46] | 0.07 [0.02; 0.18] |
| **Dysgeusia** | 0.33 [0.21; 0.48] | 0.01 [0.001; 0.15] |
| **Weight loss** | 0.27 [0.17; 0.39] | 0.03 [0.01; 0.10] |
| **Anorexia** | 0.34 [0.21; 0.50] | 0.02 [0.007; 0.04] |
| **Neutropenia** | 0.19 [0.06; 0.44] | 0.10 [0.03; 0.29] |
| **CI, confidence internal.** | | |
