# Supplementary material for: Efficacy and safety of antibody-drug conjugate combination therapy in advanced urothelial carcinoma
Source: Front Oncol. 2025 Oct 7;15:1669526. doi: 10.3389/fonc.2025.1669526 (PMC12537358; doi:10.3389/fonc.2025.1669526)
Supplement: Supplementary Table 2 — Systematic review search syntax for PubMed and Embase online libraries. [file Table2.docx]

**Supplementary Table 2. Systematic review search syntax for PubMed and Embase online libraries.**

| **Database** | **Date last searched** | **Search syntax** |
| --- | --- | --- |
| PubMed | May 2025 | ((((Urinary Bladder Neoplasms) OR (Carcinoma, Transitional Cell) OR (Urologic Neoplasms*)) AND (Immunoconjugates) OR (Enfortumab) OR (Sacituzumab) OR (Disitimab) AND ((Immunotherapy) OR (Antibodies, Monoclonal, Humanized)) |
| Embase | May 2025 | ((urinary AND ('bladder'/exp OR bladder) AND ('neoplasms'/exp OR neoplasms) OR (('carcinoma,'/exp OR carcinoma,) AND transitional AND ('cell'/exp OR cell)) OR (urologic AND neoplasms*)) AND ('immunoconjugates'/exp OR immunoconjugates) OR 'enfortumab'/exp OR enfortumab OR 'sacituzumab'/exp OR sacituzumab OR disitimab) AND ('immunotherapy'/exp OR immunotherapy OR (('antibodies,'/exp OR antibodies,) AND monoclonal, AND humanized)) |
